# Tubeimoside-2 Triggers Methuosis in Hepatocarcinoma Cells through the MKK4–p38α Axis

**DOI:** 10.3390/pharmaceutics15041093

**Published:** 2023-03-29

**Authors:** Yichao Gan, Chen Wang, Yunyun Chen, Linxin Hua, Hui Fang, Shu Li, Shoujie Chai, Yang Xu, Jiawei Zhang, Ying Gu

**Affiliations:** 1 Cancer Institute (Key Laboratory of Cancer Prevention and Intervention, China National Ministry of Education), the Second Affiliated Hospital, Zhejiang University School of Medicine, Hangzhou 310009, China; 2 Institute of Genetics, Department of Genetics, Zhejiang University School of Medicine, Hangzhou 310058, China; 3 Shanghai General Hospital, Shanghai Jiao Tong University School of Medicine, Shanghai 200025, China; 4 Department of Oncology, Ningbo First Hospital, Ningbo 315010, China; 5 Department of Hematology, the Second Affiliated Hospital, Zhejiang University School of Medicine, Hangzhou 310009, China; 6 Zhejiang Provincial Key Laboratory for Cancer Molecular Cell Biology, Life Sciences Institute, Zhejiang University, Hangzhou 310058, China; 7 Zhejiang Provincial Key Lab of Genetic and Developmental Disorder, Hangzhou 310058, China; 8 Zhejiang Laboratory for Systems & Precision Medicine, Zhejiang University Medical Center, Hangzhou 311121, China

**Keywords:** tubeimoside 2, methuosis, hepatocellular carcinoma, cholesterol biosynthesis, p38α

## Abstract

Liver cancer, consisting mainly of hepatocellular carcinoma, is the third leading cause of cancer-related mortality worldwide. Despite advances in targeted therapies, these approaches remain insufficient in meeting the pressing clinical demands. Here, we present a novel alternative that calls for a non-apoptotic program to solve the current dilemma. Specifically, we identified that tubeimoside 2 (TBM-2) could induce methuosis in hepatocellular carcinoma cells, a recently recognized mode of cell death characterized by pronounced vacuolization, necrosis-like membrane disruption, and no response to caspase inhibitors. Further proteomic analysis revealed that TBM-2-driven methuosis is facilitated by the hyperactivation of the MKK4–p38α axis and the boosted lipid metabolism, especially cholesterol biosynthesis. Pharmacological interventions targeting either the MKK4–p38α axis or cholesterol biosynthesis effectively suppress TBM-2-induced methuosis, highlighting the pivotal role of these mechanisms in TBM-2-mediated cell death. Moreover, TBM-2 treatment effectively suppressed tumor growth by inducing methuosis in a xenograft mouse model of hepatocellular carcinoma. Taken together, our findings provide compelling evidence of TBM-2’s remarkable tumor-killing effects by inducing methuosis, both in vitro and in vivo. TBM-2 represents a promising avenue for the development of innovative and effective therapies for hepatocellular carcinoma, one that may ultimately offer significant clinical benefits for patients with this devastating disease.

## 1. Introduction

Liver cancer is a nightmare for people in both developed and especially developing countries [1,2]. It has become the third leading cause of cancer-related death globally [3]. Around 80~90% of liver cancer pathologically belongs to hepatocellular carcinoma (HCC) [1]. Despite adequate medical surveillance providing a head start to handling the HCC at an early stage, it is discouraging that the majority of HCC patients are diagnosed at the advanced stage [4], with limited treatment options available thus far. Targeted therapy is the only hope for advanced-stage HCC patients. However, the benefits of standard sorafenib-based targeted therapy remain modest [5], and more frustratingly, targeted therapy inevitably leads to drug resistance in clinical practice due to cancer cell adaptions, including apoptosis resistance [6,7,8]. Consequently, there is an urgent need to develop non-apoptotic cell-death-inducing agents to augment the drug pools available for single-drug treatment or drug combinations.

Regulated cell death (RCD) is a universal biological phenomenon that plays a pivotal role in both physiological and pathological processes [9], of which its multifaceted nature presents a multitude of opportunities for the development of novel druggable targets for cancer treatment. The diverse mechanisms underlying RCD have resulted in the classification of classical RCD into several distinct forms on the basis of the fundamental aspects of each process: (1) apoptosis (both intrinsic and extrinsic ones); (2) mitochondrial permeability transition-driven necrosis; (3) autophagy-dependent cell death; (4) lysosome-dependent cell death; (5) immunogenic cell death; (6) necroptosis; (7) ferroptosis; (8) pyroptosis; (9) NETotic cell death; (10) parthanatos [10]. Among these aforementioned forms of RCD, methuosis has only recently been recognized as a distinct type of regulated cell death, being derived from macropinocytosis, and it could be induced by genetic as well as pharmacological manipulation that drives the balanced macropinocytic process into an overloaded state. The hallmark features of methuosis include swelling or severe vacuolization, necrosis-like membrane disruption, and little or no responsiveness to caspase inhibitors [11]. On the basis of the current knowledge, the key features that distinguish methuosis from other forms of RCD include the presence of large intracellular single-membrane structures and specific labeling with fluorescent macropinosome tracers, such as 70 kDa dextran dye or lucifer yellow.

Macropinocytosis, a cellular process of internalization and engulfment of extracellular fluid and particles, starts from plasma membrane ruffling, forming an actin-driven large-sized compartment named macropinosome (200 nm–5 μm in diameter). Subsequently, macropinosomes mature by heterogenous fusion with other types of endosomes and acidification by fusion with lysosome. Macropinocytosis serves as a means of nutrient scavenging as well as a built-in mechanism of drug resistance in multiple cancer types [12,13,14,15]. Oncogenic alterations such as RAS G12V mutation in cancer cells significantly intensify macropinocytosis flux, leading to the acquisition of amino acids, sugars, fatty acids, and nucleotides [16], which in turn provide crucial relief to the stress of nucleotide synthesis elicited by conventional chemotherapeutic agents. These agents specifically target enzymes essential for de novo nucleotide synthesis or cause DNA damage, thereby increasing the demand for nucleotides for DNA repair [13]. While currently most efforts have been made to block macropinocytosis to disrupt the metabolic activity of cancer cells [17,18,19], alternatively, manipulating this pathway pharmacologically to trigger methuosis may potentially offer novel insights into cancer treatment and even the drug resistance dilemma.

Traditional Chinese medicine offers great opportunities for modern medical application [20]. Tubeimosides (TBMs), including TBM-1, TBM-2, TBM-3, and TBM-4, are pharmacologically active triterpenoid saponins extracted from the traditional Chinese herb *Rhizoma Bolbostemmatis* [21]. It has recently been reported that TBMs are potent anti-tumor molecules against a broad spectrum of human cancer cell lines [21]. The molecular mechanisms reported include enhanced apoptosis, suppressed metastasis, and anti-proliferation [22,23]. However, the effects of TBMs on HCC have not been investigated. In this study, we demonstrated the potent inhibitory effect of TBM-2 on HCC cell proliferation and in vivo tumor growth by triggering methuosis. Furthermore, we identified that the activation of the MKK4–p38α axis is imperative for TBM-2-induced methuosis, concomitant with intensified lipid metabolism, especially steroid biosynthesis. Hence, our study provides a promising natural compound that may improve HCC management in the future.

## 2. Materials and Methods

### 2.1. Reagents

Antibodies against phospho-p38α (Thr180/Tyr182) (Cat# 4511S) and phospho-MKK4 (Ser257) (Cat# 4514S) were purchased from Cell Signaling Technologies (Beverly, MA, USA). Antibodies against MKK4 (Cat# A14781) were from ABclonal Technology (Wuhan, China). Antibody against p38 (Cat# sc-81621) was from Santa Cruz biotechnology (Santa Cruz, CA, USA). Antibody against GAPDH (Cat# 60004-1-Ig) was from Proteintech Group (Chicago, IA, USA). Tubeimoside-2 was purchased from Chengdu Biopurify Phytochemicals Ltd. (Chengdu, China). Small-molecular inhibitor MRT68921 (Cat# S7949) was from Selleck Chemicals(Houston, TX, USA); other small molecular inhibitors, 3-methyladenine (Cat# HY-19312), Wortmannin (Cat# HY-10197), EIPA (Cat# HY-101840), PD 169,316 (Cat# HY-10578), EHop-016 (Cat# HY-12810), and EHT 1864 (Cat# HY-16659) were purchased from MedChemExpress (MCE, Shanghai, China). We purchased 70 kDa dextran (Cat# D1818) and lucifer yellow (#L1177) from Life Technologies (Waltham, MA, USA). All reagents unless otherwise stated were purchased from Thermo Fisher Scientific (Waltham, MA, USA).

### 2.2. Cell Culture

Huh-7, JHH-7, LM3, Hep 3B, and Hep G2 cells were purchased from ATCC and cultured in Eagle’s Minimum Essential Medium (MEM) with 10% fetal bovine serum, 100 IU/mL penicillin, and 100 µg/mL streptomycin in 75 cm^2^ culture flasks. All the cells were incubated in a humidified atmosphere with 5% CO_2_ at 37 °C with medium renewal of 2–3 times a week depending on cell density. SNU387 cells was purchased from ATCC and cultured in RPMI-1640 medium with 10% fetal bovine serum, 100 IU/mL penicillin, and 100 µg/mL streptomycin in 75 cm^2^ culture flasks.

### 2.3. Cell Viability Assay

For the pharmacological inhibition of TBM-2 on HCC cell lines, procedures are as follows: cells were first sub-cultured into a 96-well plate with essentially an even number of cells in each well. A serial dilution of TBM-2 with a dilution ratio of 2, from 32 µM to 0.25 µM, replaced the medium as soon as cells were adherent. The cell survival rate assay was then performed with cells after 24 h treatment of TBM-2. Moreover, this assay was conducted with a CellTiter 96 Aqueous Cell Proliferation Kit (Promega, Madison, WI, USA). A total of 20 µL of CellTiter 96 Aqueous One Solution Reagent was placed into each well of the 96-well assay plate containing the samples in 100 µL of culture medium. For the cell survival rate assay, cells were also plated to a 96-well plate with a uniform number of each well.

### 2.4. Dextran/Lucifer Yellow Uptake Assay

Approximately 2 × 10^5^ cells were seeded to the confocal dish (#L1177, Thermo, Waltham, MA, USA)). After it was fully adherent, TBM-2 treatment was performed. Then, we rinsed these cells three times in PBS and proceeded to incubate them with either Dextran or lucifer yellow for 12 h with final concentrations of 125 µg/mL and 100 µg/mL, respectively. Images were captured by Nikon C2 plus on the basis of the manufacturer’s instructions.

### 2.5. Transmission Electron Microscopy

Cells, either treated with TBM-2 or not, were fixed with 2% glutaraldehyde in PBS, and samples were processed and embedded by the electron microscopy facility at Zhejiang University, Hangzhou. Images were taken with JEOL 1200 EX II TEM (JEOL, Tokyo, Japan) on the basis of the manufacturer’s instructions.

### 2.6. Proteomic Analysis

This was performed by the PTM-Bio Company according to the established protocols. Briefly, cells were harvested to fetch whole proteins, which were further proceeded to digestion. After the trypsin digestion procedure, peptides were labeled by TMT/iTRAQ according to the manufacturer’s protocol for TMT kit/iTRAQ kit. Using a Thermo Betasil C18 column (5 μm particles, 10 mm ID, 250 mm length), the tryptic peptides were fractionated by high pH reverse-phase HPLC. These peptides were subjected to NSI source followed by tandem mass spectrometry (MS/MS) in Q ExactiveTM Plus (Thermo) coupled online to the UPLC. Detailed settings: electrospray voltage applied was 2.0 kV; m/z scan range was 350 to 1800 for full scan; intact peptides were detected in the Orbitrap at a resolution of 70,000. Peptides were then selected for MS/MS using the following settings: Normalized Collision Energy setting as 28; the fragments were detected in the Orbitrap at a resolution of 17,500; and automatic gain control was set at 5E4. MS data were processed using Proteome Discoverer 1.3.

### 2.7. Western Blot

Cells were lysed in M-PER Mammalian Protein Extraction Reagent with Protease Inhibitor Cocktail and EDTA, according to the instruction of Thermo Fisher Scientific. Measurement of protein concentration was performed by using Pierce BCA Protein Assay Kit. A uniform number of proteins of differently treated cells were separated by 10% SDS–PAGE and transferred to the polyvinylidene difluoride (PVDF) membrane. The membrane was blocked with 5% nonfat milk in TBS buffer containing 0.1% (*v*/*v*) Tween-20 (pH 7.5) and then incubated overnight at 4 °C with primary antibody (1:1000 dilution), followed by incubation with HRP-conjugated secondary antibody (1:1000 dilution) at room temperature for 1 h. ECL Plus Western Blotting Substrate was used to visualize protein signals.

### 2.8. Total Cholesterol Assay

Cells, either treated with TBM-2 or DMSO for 24 h, were harvested and then lysed in 2% TritonX-100 for 30 min. Total cholesterol was able to be detected by adding working solution from the total cholesterol assay kit (Nanjing Jiancheng Bioengineering institute).

### 2.9. Xenograft Experiments

The animal experiment adhered to the principles of care and use of laboratory animals and was approved by the Institutional Animal Care and Use Committee of Zhejiang University, China. Female BALB/c nude mice were purchased from SHANGHAI SLAC LABORATORY ANIMAL CO. LTD. (Shanghai, China). Hep 3B cells (approximately 2.5 × 10^6^ cells) in 100 μL PBS were subcutaneously inoculated into the right flank of 5-week-old female BALB/c nude mice. Treatment was initiated when tumors reached 80 mm^3^. The drug efficacy study was performed with two groups according to different doses of TBM-2, including control and TBM-2 (4 mg/kg/day, *i.p.*; n = 5/group). Animals were randomized to receive control solvent and TBM-2. The equation used to evaluate the tumor volume:

$$\mathrm{Tumor}\;\mathrm{volume}{\left(\mathrm{mm}\right)}^{3}=(\mathrm{length}\times {\mathrm{width}}^{2})\times 0.52$$

### 2.10. Statistical Analysis

All experiments, except for statistical analysis, were randomized and blinded. Three to five independent repeat experiments were carried out throughout our whole project. All statistical analyses were conducted using GraphPad Prism version 7.0 (GraphPad Software Inc., La Jolla, CA, USA).

## 3. Results

### 3.1. TBM-2 Treatment Induced Methuosis in Hepatocarcinoma Cells

**Figure 1:**
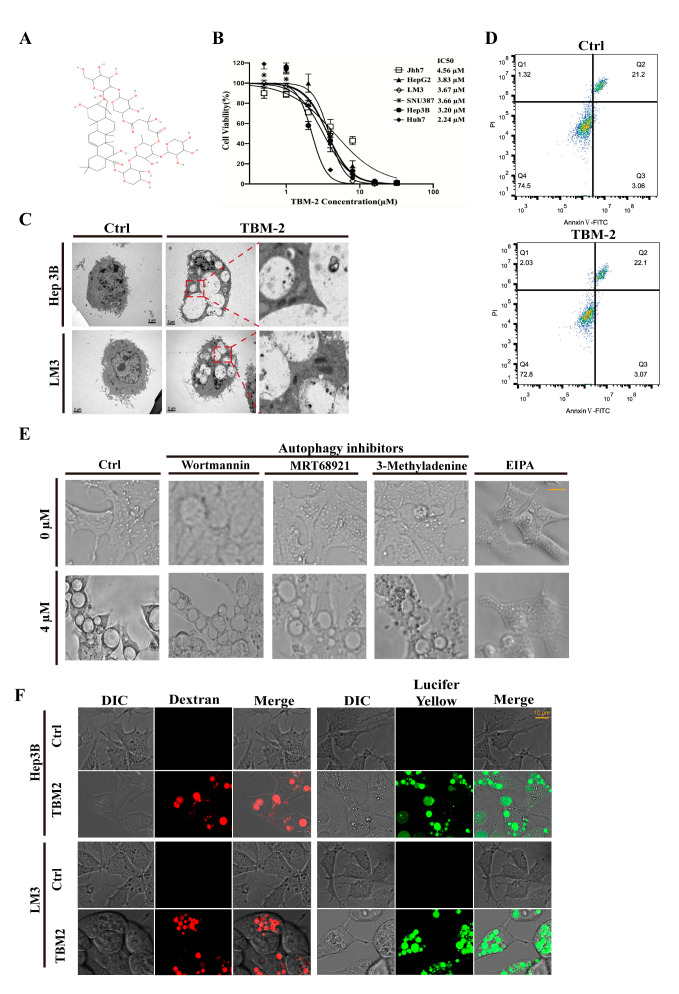
TBM-2 treatment induced methuosis in hepatocarcinoma cells. (**A**) Two-dimensional structure of TBM-2. (**B**) Cell viability curve of six HCC cell lines treated with serial dilution of TBM-2 for 24 h. A total of 1.5 × 10^3^ cells were plated in complete growth medium in 96-well plates, and MTS assay was performed to assess cell viability. (**C**) Extraordinary vesicles with a single membrane were visualized by TEM induced by 4 µM TBM-2 for 24 h in both LM3 and Hep 3B. (**D**) Cells were treated with either DMSO or 2 µM TBM-2 for 24 h and then stained with annexin V/propidium iodide (PI) for flow cytometric analysis. (**E**) After 24 h TBM-2 treatment on Hep 3B, vacuolization appeared in those pretreated with autophagy inhibitors (3-methyladenine, Wortmannin, and MRT68921) for 3 h, while it was barely observed in those pretreated with the macropinocytosis inhibitor, EIPA, for 3 h. Scale bars, 10 µm. (**F**) Cells either treated with DMSO or TBM-2 for 24 h were further incubated with macropinocytosis tracer, dextran, and lucifer yellow for 12 h, with final concentrations of 125 µg/mL and 100 µg/mL, respectively. Scale bars, 10 µm.

TBM-2, as one of the pharmacologically active components extracted from traditional Chinese herbs, has recently been reported as a potent anti-tumor molecule against several human cancer cell lines [21] (Figure 1A). To evaluate the inhibitory effect of TBM-2 on hepatocarcinoma cells, we first carried out a cell viability assay on six HCC cell lines: Hep 3B, Jhh-7, LM3, SNU387, Huh7, and Hep G2. All HCC cell lines showed a remarkable response to TMB-2 treatment with an IC50 range from 2.24 µM to 4.56 µM (Figure 1B). Intriguingly, extra-large vacuoles appeared in these HCC cells during TBM-2 treatment. Due to the most apparent vacuoles in LM3 and Hep 3B cells after TBM-2 treatment, we took LM3 and Hep 3B cell lines for further study. These impressive vesicles triggered by TBM-2 were encapsulated by a single-layer membrane rather than a bilayer membrane, as determined by transmission electron microscope (TEM) scanning (Figure 1C). As far as we know, phagosomes, similar with these large vesicles, only belong to specific cells [24]. Thus, we speculated that these single-layer membrane vacuoles might be either aberrant macropinosomes or dysfunctional autolysosomes. To determine whether these vesicles are auto-lysosomes, we introduced a flow cytometric assay with annexin V/propidium iodide (PI) staining to determine apoptosis and found that basal levels of apoptosis were present in the control group, while TBM-2 treatment did not enhance apoptosis (Figure 1D). Meanwhile, we also tried three autophagy inhibitors (3-methyladenine, Wortmannin, and MRT68921) to block the formation of the autolysosome. None of the three autophagy inhibitors prevented the formation of large vesicles when treated in combination with TBM-2 (Figure 1E). Further, we labelled Hep3B and LM3 cells after TBM-2 treatment with two specific macropinosome probes, fluorescent 70 kDa dextran dye and lucifer yellow [25,26], respectively. Moreover, these vesicles were, indeed, positively labelled by these two markers separately (Figure 1F), suggesting that these vesicles are derived from macropinosome. Moreover, EIPA, which specifically inhibits macropinocytosis rather than any other endocytosis processes, confers Hep 3B resistance to TBM-2 induced vacuolization (Figure 1E). Hence, we demonstrated that TBM-2 specifically triggers aberrant macropinocytosis, or methuosis as it is called, in hepatocarcinoma cells.

### 3.2. TBM-2 Enhanced Lipid Metabolism to Support Methuosis

**Figure 2:**
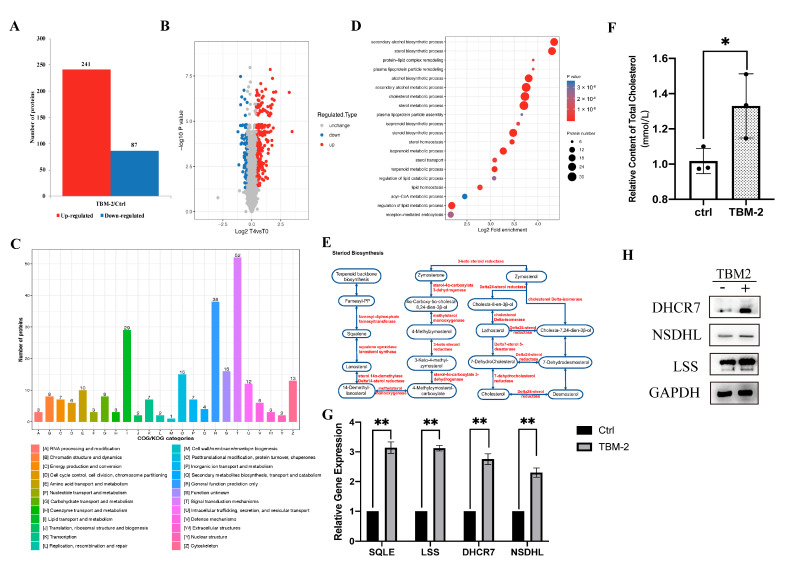
TBM-2 perturbated the signal transduction pathway and intensified lipid metabolism. (**A**) Numbers of up- and downregulated proteins identified from Hep 3B cells after 4 µM TBM-2 treatment. (**B**) Volcano plot in protein abundance with foldchange >1.3 as the threshold of significant upregulation and foldchange <1/1.3 as significant downregulation. T4 represents 4 µM TBM-2 treatment, and T0 refers to negative control. (**C**) Comprehensive COG functional classification indicates significant upregulation of signal transduction and lipid transport and metabolism-related proteins. (**D**) GO enrichment analysis of upregulated proteins showed enrichment of the lipid metabolism pathways. (**E**) As a representative lipid metabolism pathway, almost all regulatory enzymes of the steroid biosynthesis pathway were upregulated (colored as red) due to TBM2 treatment. (**F**) Hep 3B was either treated with DMSO or 2 µM TBM-2 for 24 h, and their total cholesterol level was extracted for quantification by the Total cholesterol assay kit. (**G**,**H**) The gene expression level (**G**) and protein level (**H**) of representative steroid biosynthesis enzymes between groups treated with DMSO and 2 µM TBM-2 for 24 h. Data represent the mean ± SD of three independent experiments. * *p* < 0.05, ** *p* < 0.01.

Macropinocytosis is typically characterized by a homeostatic equilibrium, whereby it satisfies nutrient-scavenging demands while exhibiting a limited proclivity towards the pathway of methuosis. To clarify intrinsic pathways or components in HCC cell lines that were affected by TBM-2, we performed a proteomic analysis on Hep 3B cells treated with 4 µM TBM-2 for 24 h. Overall, 6207 quantifiable proteins were identified, of which, 241 proteins were upregulated, while 87 proteins were downregulated (*p* value < 0.05) due to TBM-2 treatment (Figure 2A). A relative standard deviation (RSD) method was employed to endorse the quality of our data (Figure S1). Significant up- and downregulated proteins were identified with foldchange >1.3 and foldchange <1/1.3, respectively (Figure 2B). Further, we mapped those up- and downregulated proteins to Clusters of Orthologous Groups of protein (COG) categories. As determined by COG analysis, proteins were mostly enriched in categories of function of signal transduction, general function prediction only, and lipid transport and metabolism (Figure 2C). Meanwhile, GO enrichment analysis of these up- and downregulated proteins also showed the same enriched processes (Figure S2). When we focused on upregulated proteins, the lipid-metabolism-related processes became far more remarkable, with almost all the top 20 biological processes related to lipid metabolism (Figure 2D), indicating that TBM-2 may regulate proteins that participate in rewiring the lipid metabolic pathway. Specifically, all the enzymes in steroid biosynthesis were essentially upregulated, as shown in the quantitative proteomic data (Figure 2E). Moreover, the key enzymes in the steroid biosynthesis pathway were indeed upregulated at both protein levels and mRNA levels, including DHCR7, NSDHL, and LSS (Figure 2G,H). Accordingly, total cholesterol was indeed increased in the TBM-2 treatment group compared to the control group (Figure 2F). Thereinto, steroid biosynthesis upregulation was in accordance with the dependence of methuosis on steroids represented by cholesterol, which is necessary for the ruffling of the plasma membrane and the initiation of micropinocytosis [27,28]. Meanwhile downregulated proteins were mostly involved in cell motility and migration (Figure S3). In conclusion, TBM-2 treatment remarkably upregulated lipid metabolism, especially the cholesterol biosynthesis process, to support methuosis.

### 3.3. TBM-2 Treatment Hyperactivated the MAPK Signal Pathway

**Figure 3:**
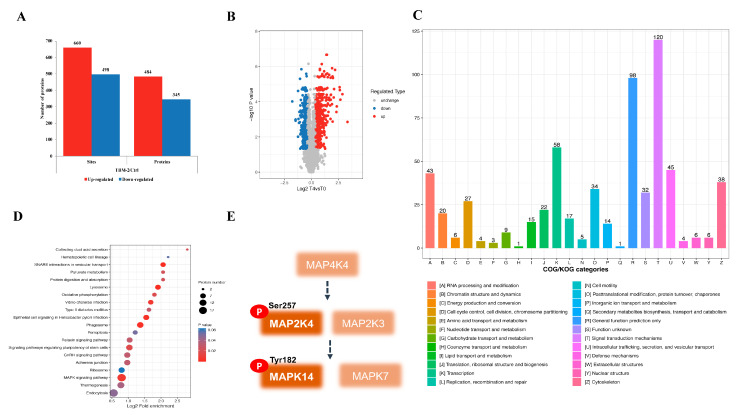
Phospho-proteomic analysis revealed the MAPK signal pathway. (**A**) Numbers of up- and downregulated proteins and phosphorylation sites after TBM-2 treatment. (**B**) Alterations in the phosphorylation level demonstrated as volcano plots with log2-transformed changes in protein abundance. T4 represents 4 µM TBM-2, and T0 refers to ctrl. (**C**) Comprehensive COG functional classification of phosphorylated proteins indicated significant upregulation of signal transduction, transcription and intracellular trafficking, secretion, and vesicular transport. (**D**) GO enrichment analysis of upregulated phosphorylated proteins surfacing from the MAPK signal pathway. (**E**) MAPK signal proteins that were detected with upregulated phosphorylation changes in functional sites.

Given the critical role of MAPK or PI3K signal pathways in macropinocytosis [29,30], we decided to perform phospho-proteomic analysis to explore the exact pathway perturbed by TBM-2 to tilt the balanced macropinocytosis cycle to methuosis. In total, 3848 quantifiable proteins were identified, among which 484 proteins were subjected to an upregulated state of phosphorylation, while 345 proteins were subjected to a downregulated state of phosphorylation. Moreover, 660 phosphorylated sites were determined as upregulated, while 498 phosphorylated sites were downregulated (Figure 3A). We plotted the significantly modified proteins due to TBM-2 treatment with the foldchange >1.3 as significant upregulation and foldchange <1/1.3 as significant downregulation (Figure 3B). Furthermore, RSD analysis of the different treatment groups supported the reliability in terms of statistics (Figure S4). In consistency with proteomic data, signal transduction mechanisms and general function prediction were only ranked as the top two functional processes, as indicated by COG analysis (Figure 3C). Moreover, transcription, intracellular trafficking, secretion, and vesicular transport, as well as RNA processing and modification, were also on the top list of COG classification. These functional processes supported the global perturbation at a proteomic level, especially the expression of enzymes involved in lipid metabolism, as well as overloaded macropinosomes that led to methuosis due to TBM-2 treatment. On the basis of the GO enrichment analysis on proteins with an up-/downregulated status on phosphorylation, the enzyme-linked receptor protein signaling pathway may largely account for such a phenotype (Figure S5). Further interrogation on proteins with upregulated phosphorylation status found that the MAPK signal pathway turned out to play a critical role (Figure 3D). There were five built-in components of this pathway that were highly phosphorylated: MAPK7, MAPK14 (p38α), MAP2K3 (MKK3), MAP2K4 (MKK4), and MAP4K4 (Figure 3E). Together, these results indicated that the MAPK signal pathway was hyperactivated after TBM-2 treatment and might be involved in TBM-2 induced methuosis through enhancing lipid metabolism.

### 3.4. TBM-2 Induced Methuosis via the MKK4–p38α Axis

**Figure 4:**
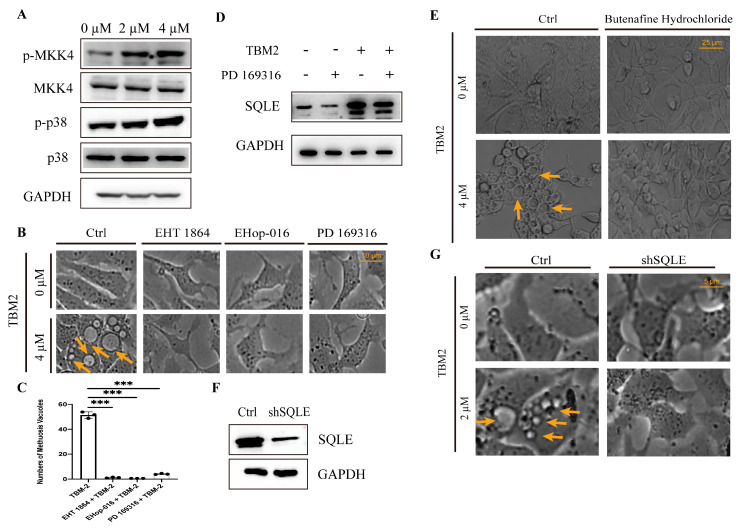
The MKK4–p38α axis mediated methuosis induced by TBM-2. (**A**) Representative Western blots of the MKK4–p38α axis in the TBM-2 treatment group and control group. Cells were cultured in equal numbers in a 6-well plate, treated for 24 h with indicated concentrations, and harvested for Western blot assay. (**B**) Morphological changes of cells that were pre-treated with the Rac1 inhibitors, namely, 5 µM EHop-016 and 20 µM EHT 1864, or the p38α inhibitor, 200 µM PD 169316, before being treated with TBM-2 for 24 h. Arrows indicate methuosis, Scale bars, 10 µm. (**C**) Number of methuosis vacuoles in cells that were pre-treated with the Rac1 inhibitors, namely, 5 µM EHop-016 and 20 µM EHT 1864, or the p38α inhibitor, 200 µM PD 169316, before being treated with TBM-2 for 24 h. *** *p* < 0.001 (**D**) Representative Western blots of squalene epoxidase, a key rate-limiting enzyme in cholesterol biosynthesis, after different treatments for 24 h. Concentrations of TBM-2 and PD 169,316 were 2 µM and 200 µM, respectively. (**E**) Phenotypes of cells that were pretreated with 200 µM squalene epoxidase inhibitor, butenafine hydrochloride, or DMSO and subsequently treated with TBM-2 for 24 h. Arrows indicate methuosis. (**F**) Western blots for validation of the knock-down of squalene epoxidase. (**G**) Phenotypes of cells with different protein levels of squalene epoxidase that were treated with or without TBM-2 for 6 h. Arrows indicate methuosis.

The MAPK pathway proteins with upregulated phosphorylation status were essentially enriched to p38 cascade: p38α and its direct upstream regulators MKK3 and MKK4. MKK4 and p38α have been reported to be involved into macropinocytosis or methuosis [31,32,33]. Moreover, the activation of p38α through phosphorylation on its Tyr182 by MKK4 was determined [34]. Therefore, we first confirmed the phospho-activation of these two proteins after TBM-2 treatment by Western blot assay (Figure 4A). Conversely, pharmacologically inhibiting p38α with PD 169,316 suppressed the methuosis induced by TBM-2 (Figure 4B,C). Due to the lack of commercial MKK4 inhibitors as well as there being barely no phosphorylation alteration in the kinase pockets of upstream MAPKs in our phospho-proteomic data, we managed to pharmacologically inhibit a reported upstream regulator of MKK4, Rac1 [35]. We introduced two small molecular inhibitors of Rac1, namely, EHop-016 and EHT 1864, and the result demonstrated that pharmacological inhibition of Rac1 suppressed methuosis triggered by TBM-2 in Hep 3B cells (Figure 4B,C). Besides, other inhibitors of MKK4–p38α axis were also used to validate the functional role of this axis in methuosis (Figure S6). Given the supportive role of steroid biosynthesis in methuosis induced by TBM-2, as well as a previous report about the transcriptional regulation on *SQLE* by a p38 inhibitor, PD169316 [36], we hypothesized that the MKK4–p38α axis would up-regulate SQLE to support steroid biosynthesis. As we blocked the activity of p38α, we observed the declined protein level of SQLE compared to the TBM-2 single-treatment group (Figure 4D). Moreover, pharmacological inhibition of SQLE with butenafine hydrochloride and knock-down of SQLE both successfully rescued methuosis induced by TBM-2 (Figure 4E–G). Taken together, we proposed that the MKK4–p38α axis may be the molecular underpinning of TBM-2-induced methuosis.

### 3.5. TBM-2 Triggered Methuosis and Suppressed Hepatocarcinoma Tumor Growth In Vivo

**Figure 5:**
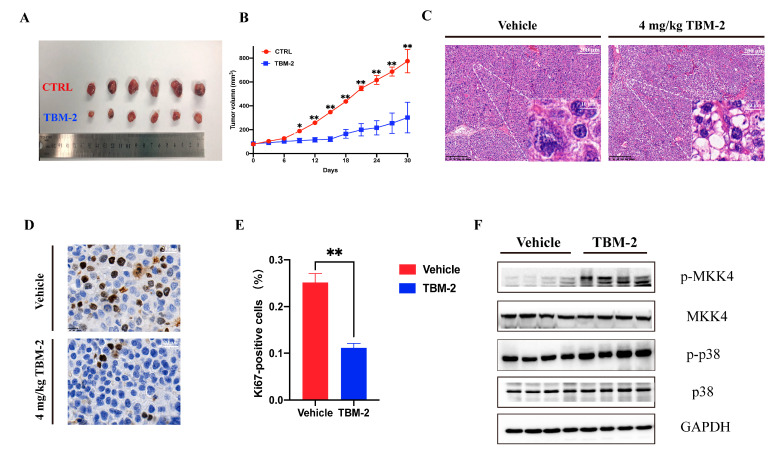
TBM-2 triggered methuosis in hepatocarcinoma tumors in vivo. (**A**) Representative tumors excised from vehicle- or TBM-2-treated animals (n = 6). TBM-2 or vehicle was delivered by intraperitoneal administration of 4 mg/kg/day. (**B**) Tumor volumes that were measured every 3 days after TBM-2 or vehicle treatment (n = 6 mice per group). (**C**) H&E staining indicated pathological vacuolization in the TBM-2 treatment group. Scale bars, 200 µm and 10 µm, respectively. (**D**) Ki67 staining confirmed the inhibitory effect of TBM-2 on the growth of hepatocarcinoma tumor in vivo. Scale bar, 20 µm. (**E**) Difference of Ki67 positive rate between vehicle and TBM-2 treatment group. (**F**) Protein levels of tumor samples from vehicle and TBM-2 treatment groups. The data represent the mean ± SD of three independent experiments. * *p* < 0.05, ** *p* < 0.01.

To assess the anti-tumor effect in vivo, Hep 3B cells were subcutaneously inoculated into the right flank of 5-week-old female nude mice (approximately 2.5 × 10^6^ cells per mice). TBM-2 or control vehicle was provided by intraperitoneal administration of 4 mg/kg/day to these mice. The tumor size was measured every 3 days after the injection of TBM-2 or vehicle. At the endpoint, retarded tumor growth was significantly manifested in TBM-2-treated mice (Figure 5A,B). Moreover, large vacuoles of methuosis appeared in H&E (hematoxylin-eosin staining) staining (Figure 5C). Further Ki67 staining confirmed the inhibitory effect of TBM-2 on the growth of hepatocarcinoma tumor in vivo (Figure 5D,E). Furthermore, the phosphorylation status of both Thr-180 and Tyr-182 of p38α were upregulated, as the Western blot indicated (Figure 5F). These results demonstrated a potent anti-tumor effect of TBM-2.

## 4. Discussion

HCC is one of the leading causes of cancer-related mortality worldwide. Despite extensive efforts to develop therapeutic strategies, its treatment remains unsatisfied. Targeted therapy, represented by sorafenib, is the mainstay for HCC treatments [5]; however, it only confers modest benefits and ultimately leads to drug resistance in clinical practice, primarily due to apoptosis resistance [6,7,8]. Consequently, innovative approaches targeting critical nodes that induce non-apoptotic cell death have been proposed as promising strategies to address the current treatment dilemma.

Among these, methuosis has emerged as a new member of the list of non-apoptotic cell death programs that could potentially shed new light on the management of HCC. Cancer cells, particularly those with constitutive activated Ras signals, could have constitutively active macropinocytosis to meet the greedy nutrient demand, compared to normal cells. In other words, these cancerous cells exhibit a relatively higher dependence on macropinocytosis than their normal counterparts. Therefore, leveraging on such dependency may selectively kill cancer cells while sparing normal cells. Hijacking macropinocytosis through pharmacological manipulation to render cancer cell overloaded, thereby triggering methuosis, is an attractive alternative for treating HCC. Steroid biosynthesis, an essential biological process, plays a crucial role in supporting methuosis. It has been evidenced that cholesterol is vital for the stimulation of membrane ruffling and macropinocytosis [27]. Furthermore, steroid biosynthesis is highly active in the liver, making HCC cells more susceptible to pharmacological perturbations that induce methuosis. Therefore, the manipulation of steroid biosynthesis to trigger methuosis represents a promising approach to treating HCC [37].

In the present study, we documented the effectiveness of TBM-2, a compound derived from a traditional Chinese herb, as an anti-tumor agent for hepatocellular carci-noma (HCC) cell lines. Our findings reveal that the mechanism underlying TBM-2’s thera-peutic potential is the MKK4–p38α axis, which triggers the process of methuosis, resulting in cell death. To our knowledge, this is the first report of the crucial role of the MKK4–p38α axis in methuosis, which hijacks macropinocytosis to disrupt cellular homeostasis and ultimately leads to cell death. Furthermore, we identified that lipid metabolism is a key participant in methuosis induced by TBM-2, where the rate-limiting enzyme in cholesterol biosynthesis, SQLE, is regulated by p38α. Pharmacological inhibition and knock-down of SQLE both successfully rescued methuosis induced by TBM-2. Therefore, the present study not only identified a novel mechanism of anti-tumor activity for TBM-2 but also shed light on the importance of lipid metabolism in the regulation of methuosis. Moreover, we evaluated the efficacy of TBM-2 treatment in a hepatocellular carcinoma xenograft mouse model, where we observed a significant suppression of tumor growth through the induction of methuosis. Overall, our study underscores the therapeutic potential of TBM-2 for the treatment of HCC and highlights the importance of the MKK4–p38α axis and lipid metabolism, especially cholesterol biosynthesis, in the process of methuosis.

Different from conventional strategies aimed at inducing cancer cell death by suppressing macropinocytosis, here, we propose an alternative approach to eliminate malignant cells by exploiting this process and redirecting it towards methuosis. Therefore, on the basis of this innovative mechanism, we discovered that TBM-2 exhibits a remarkable inhibitory effect on cell viability in hepatocellular carcinoma (HCC), both in vitro and in vivo, advancing it as a highly promising candidate for future application in HCC management. In the future, it is necessary to investigate the direct molecular targets of TBM-2 that are involved in methuosis. It is essential to fully elucidate the complex interplay between these targets and the MKK4–p38α axis. By doing so, we can gain a deeper understanding of the underlying molecular mechanisms that drive the anticancer effects of TBM-2 and pave the way for the development of more effective and targeted therapies for HCC.

## Data Availability

The data can be shared up on request.

## Supplementary Materials

The following supporting information can be downloaded at https://www.mdpi.com/article/10.3390/pharmaceutics15041093/s1. Figure S1: RSD of the proteomic analysis among different treatment groups; Figure S2: Gene Ontology enrichment analysis of up- and down-regulated proteins due to TBM-2 treatment; Figure S3: Gene Ontology enrichment analysis of down-regulated proteins in whole protein level due to TBM-2 treatment; Figure S4: RSD of the phospho-proteomic analysis among different treatment groups; Figure S5: GO enrichment analysis on proteins with up/down-regulated status on phosphorylation due to TBM-2 treatment; Figure S6: Cross-validation to support the functional role of MKK4–p38α axis with other inhibitors.
