# Supplementary material for: Tubeimoside-2 Triggers Methuosis in Hepatocarcinoma Cells through the MKK4–p38α Axis
Source: Pharmaceutics. 2023 Mar 29;15(4):1093. doi: 10.3390/pharmaceutics15041093 (PMC10142215; doi:10.3390/pharmaceutics15041093)
Supplement: Supplementary file 1 [file pharmaceutics-15-01093-s001.zip › pharmaceutics-2297590-supplementary.pdf]

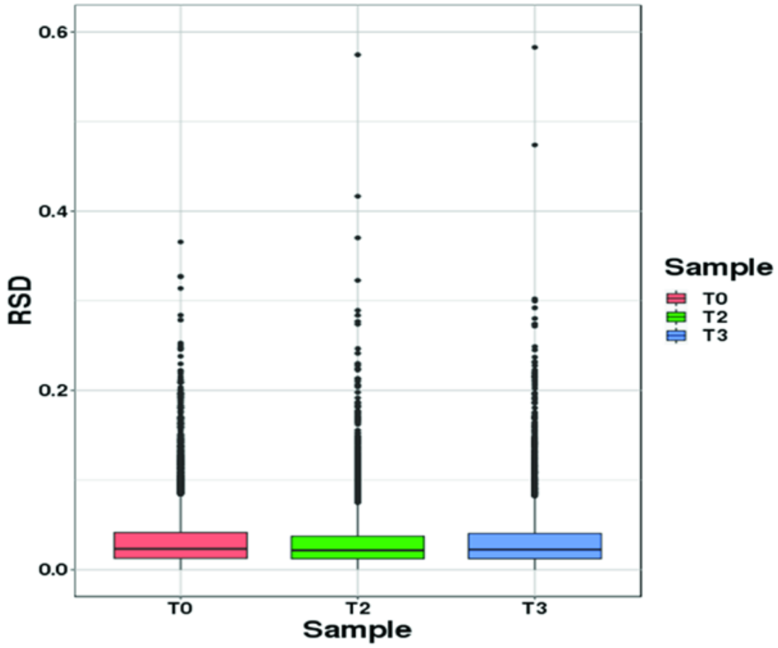

Supplement Figure1. RSD of the Proteomic Analysis among Different Treatment Groups
Cells were treated with DMSO or TBM-2 for 24h. (T0: DMSO; T2: 2μM TBM-2; T4: 4μM TBM-2)

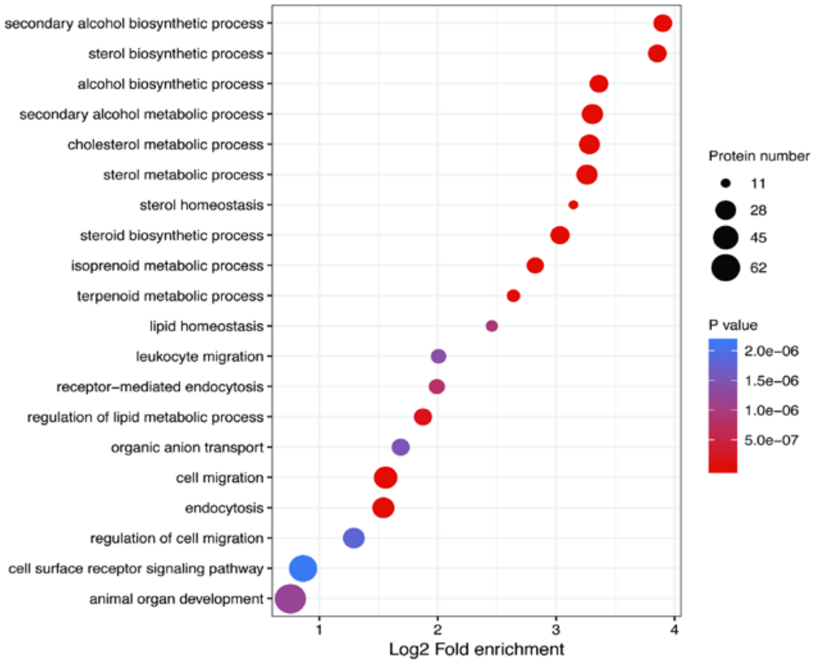

Supplement Figure2. Gene Ontology(GO) Enrichment Analysis of Up- and Down- regulated Proteins Due to TBM-2 Treatment
GO enrichment analysis of differentially regulated proteins between DMSO and TBM-2 treatment groups. Cells were equally cultured and proceeded to either DMSO or 2μM TBM-2 for 24h before harvesting.

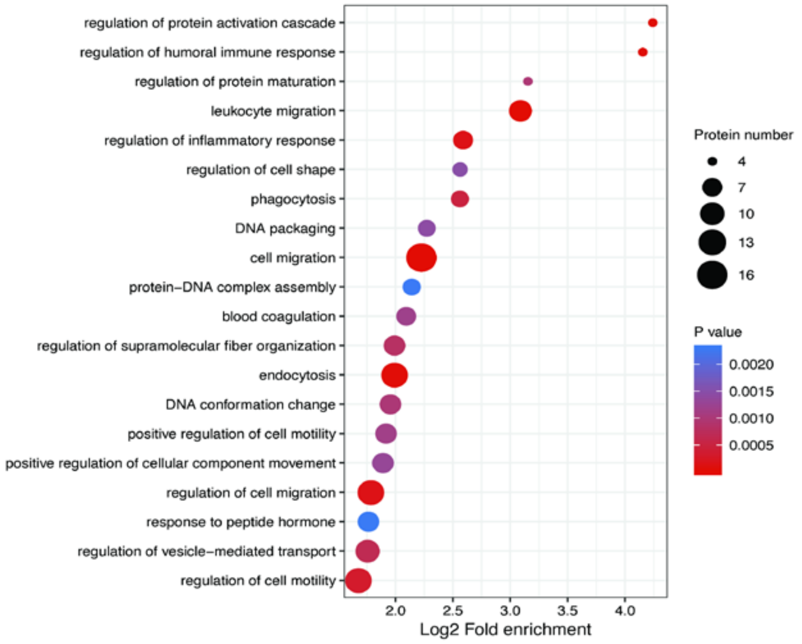

Supplement Figure3. Gene Ontology(GO) Enrichment Analysis of Down- regulated Proteins in Whole Protein Level Due to TBM-2 Treatment

GO enrichment analysis of down-regulated proteins between DMSO and TBM-2 treatment groups.
Cells were equally cultured and proceeded to either DMSO or 2μM TBM-2 for 24h before harvesting.

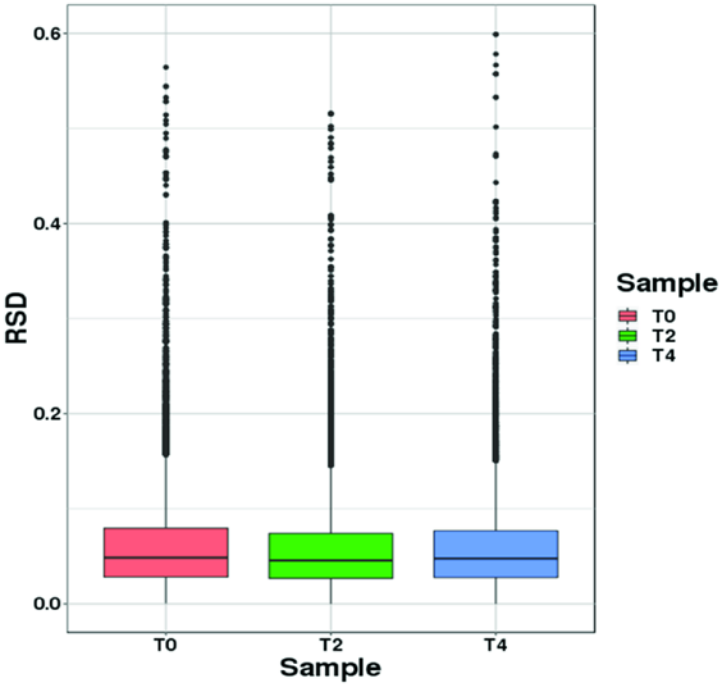

Supplement Figure4. RSD of the Phospho-proteomic Analysis among Different Treatment Groups

Cells were treated with DMSO or TBM-2 for 24h. (T0: DMSO; T2: 2μM TBM-2; T4: 4μM TBM-2)

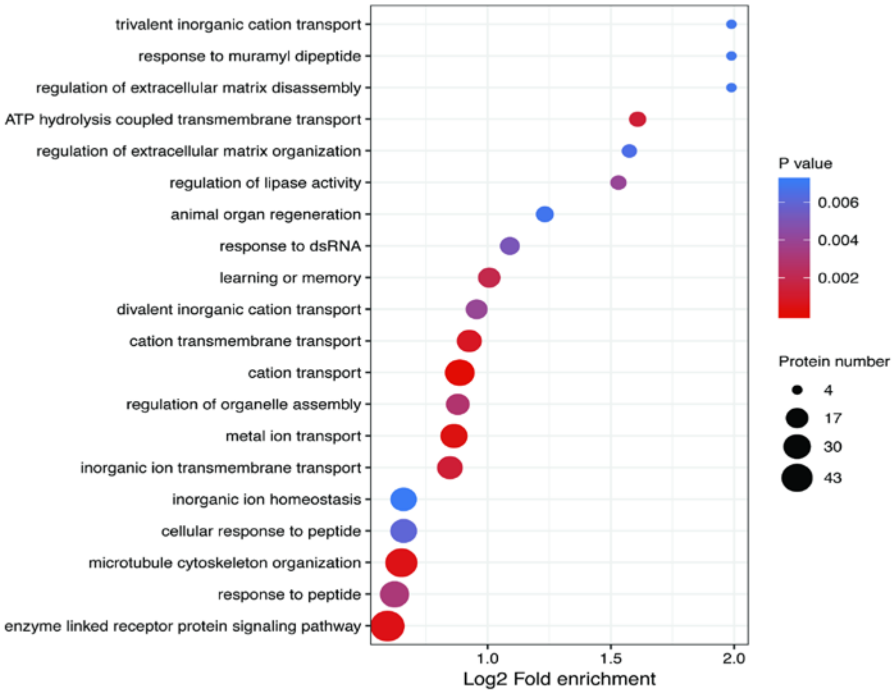

Supplement Figure5. GO Enrichment Analysis on Proteins with Up/Down-regulated Status on Phosphorylation Due to TBM-2 Treatment

Cells were treated with either DMSO or 2μM TBM-2 for 24h before proteomic analysis.

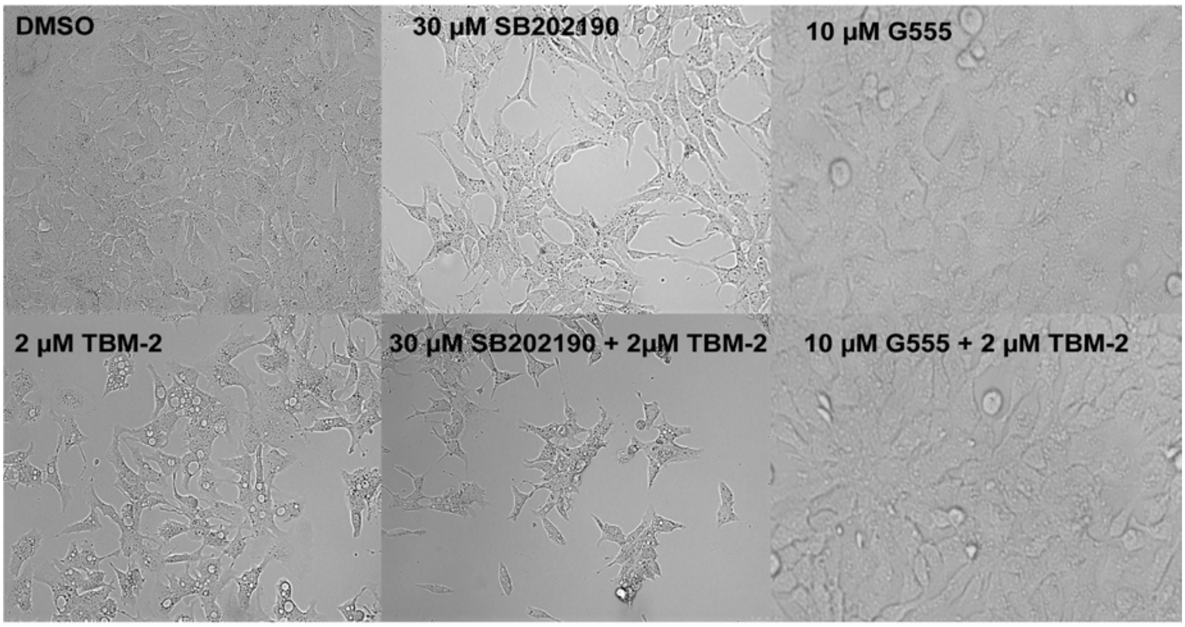

Supplement Figure6. Cross-validation to support the functional role of MKK4--p38α axis with other inhibitors.

Vacuoles were barely found in the cells that were pre-treated with 10 μM G555 (Pak1 inhibitor) or 30μM SB 202190 (p38α inhibitor) for 3h. In contrast, TBM-2 treatment alone could evoke massive vacuoles.
